# The Lung-Brain Axis in Chronic Obstructive Pulmonary Disease-Associated Neurocognitive Dysfunction: Mechanistic Insights and Potential Therapeutic Options

**DOI:** 10.7150/ijbs.109261

**Published:** 2025-05-15

**Authors:** Xiao Yu, Hui Xiao, Yushan Liu, Zhiyong Dong, Xiaoting Meng, Fang Wang

**Affiliations:** 1 Department of Histology & Embryology, College of Basic Medical Sciences, Jilin University, Changchun, 130021, China;; 2 The Key Laboratory of Pathobiology, Ministry of Education, College of Basic Medical Sciences, Jilin University, Changchun, 130021, China;; 3 Department of Forensic Medicine, Basic Medical College, Jilin University, Changchun, 130021, China;; 4 College of Basic Medical Sciences, the Medical Basic Research Innovation Center of Airway Disease in North China, Key Laboratory of Pathobiology, Ministry of Education, Jilin University, Changchun, 130021, China.

**Keywords:** Lung-brain axis, Chronic obstructive pulmonary disease (COPD), Neurocognitive disorders, COPD-related neurocognitive disorders (COPD-NCDs), Inflammation, Oxidative stress.

## Abstract

Chronic obstructive pulmonary disease (COPD) ranks as the third leading cause of global mortality, affecting 210 million individuals worldwide. Notably, 60% of COPD patients experience comorbid neurocognitive disorders. Importantly, patients with neurocognitive dysfunction often exhibit poor adherence to therapeutic interventions and medications, exacerbating their COPD morbidity and increasing hospitalization rates and mortality risk. This review explores the potential lung-brain axis in COPD, emphasizing that oxidative stress and inflammatory responses in the lungs can spread to the systemic circulation, thereby regulating in the blood-brain barrier (BBB) permeability and contributing to brain dysfunction. In addition, the role of hormone-based hypothalamic-pituitary-adrenal (HPA) axis in COPD progression is discussed. These cascading events can lead to neuronal deficits, altered glial cell function, and subsequent cognitive dysfunction. Furthermore, we provide a comprehensive overview of potential medications for treating COPD and its associated cognitive deficits, with a specific focus on anti-inflammatory and antioxidant therapies. This compilation serves as a pivotal foundation for the prevention and management of cognitive dysfunction in COPD.

## 1. Introduction

Chronic obstructive pulmonary disease (COPD) is a common respiratory disease characterized by emphysema, mucus hypersecretion, and persistent lung inflammation. Its inflammatory features include infiltration of airways and lung tissue by inflammatory cells such as neutrophils, macrophages and lymphocytes^1^. Clinically, COPD manifests as chronic airflow obstruction, accompanied by symptoms such as dyspnea, persistent cough, sputum and fatigue. It is a serious threat to human health and affects the quality of life of patients^2^. Among the various risk factors, smoking is one of the primary pathogenic contributors to COPD. Harmful particles in cigarette smoking (CS) damage the lung epithelium, resulting in increased mucin concentration and decreased IgA secretion^3^. This impairment allows bacteria to invade the airway epithelium, triggering the aggregation of macrophages, neutrophils, and lymphocytes^4^. These cells release pro-inflammatory mediators, which further recruit leukocytes, creating a vicious cycle of persistent lung inflammation^5^. Additionally, the excessive accumulation of lung macrophages and neutrophils results in elevated production of reactive oxygen/nitrogen species (ROS/RNS) and accelerates oxidative stress^6^, ^7^. CS-induced inflammation and oxidative stress in the lungs can "spill-over" into the systemic circulation, contributing to the development of chronic comorbid conditions (also known as comorbidities)^8^. These comorbidities include cardiovascular disease, osteoporosis, metabolic syndrome, mental health disorders and cognitive impairment^9^, ^10^.

Recent clinical studies have shown that up to 60 percent of COPD patients experience cognitive dysfunction, including impairments in working memory^11^, ^12^, executive function^11^, attention^13^, and delayed recall^14^. Furthermore, the prevalence of anxiety and depression in COPD patients ranges from 40 to 64% and 25 to 67%, respectively^15^-^17^. These findings suggest an intrinsic link between cognitive dysfunction and respiratory function. Studies indicate that the prevalence of cognitive decline and mood disorders in COPD patients is typically two to three times higher than in healthy individuals^18^. Importantly, neurocognitive dysfunction is associated with increased disease burden, healthcare utilization, and costs. Patients with neurocognitive dysfunction often exhibit poor adherence to therapeutic interventions and medications. This non-adherence exacerbates COPD morbidity, leading to higher hospitalization rates and mortality risk^19^-^21^. However, the pathophysiology linking COPD to neurocognitive disorders remains poorly understood, and the interaction between respiratory system and central nervous system (CNS) lacks a clear and systematic explanation. Consequently, there is currently no effective treatment for COPD-related neurocognitive disorders (COPD-NCDs)^22^-^24^.

It is widely hypothesized that insufficient airflow in COPD lungs leads to the development of hypoxia^25^, which may impair the function of enzymes responsible for the production of neurotransmitters such as dopamine and serotonin^26^, ^27^. However, COPD is a complex and highly heterogeneous disease that may affect CNS dysfunction through multiple mechanisms. For example, CS may alter brain structure and function by increasing the cumulative risk of cardiovascular disease^28^. In some cases, CNS dysfunction, such as mood disorders, may also be linked to the psychological burden of chronic illness^29^.

Recent research has highlighted the critical role of inflammation in the decline of respiratory system function in COPD. Spill-over of inflammatory factors from the lungs is thought to drive extrapulmonary complications in COPD patients. Compared to healthy individuals, COPD patients exhibit significantly elevated levels of inflammatory mediators such as c-reactive protein (CRP), interleukin (IL)-6, IL-8, fibrinogen, and tumor necrosis factor (TNF)-α in their serum^30^, ^31^. Traditionally, the brain has been viewed as an isolated system protected by the blood-brain barrier (BBB). However, emerging evidence has identified multiple pathways and factors that facilitate immune communication between the blood and the brain^32^, ^33^. For instance, inflammatory cytokines such as IL-6, TNF-α, and IL-1β can breach the BBB, disrupting the CNS and triggering neuroinflammation. This neuroinflammation can structurally damage the neurons themselves and impair neuronal function, potentially leading to COPD-NCDs^34^. In addition, some studies point to a link between CS-induced neurocognitive disorders and microglia-mediated neuroinflammation^35^. Microglia, the resident immune cells of the brain, play a crucial role in regulating neurogenesis, synaptogenesis, and cognition. However, chronic activation of microglia promotes neuronal and axonal loss, ultimately leading to neurocognitive dysfunction^36^, ^37^. Moreover, activated microglia can produce ROS, leading to oxidative stress in COPD and persistent neurocognitive dysfunction^38^. Despite these insights, the mechanisms to reverse such neuroinflammation remain unclear.

This article summarizes the brain dysfunction and neurodegenerative diseases closely related to COPD and explores the potential lung-brain axis from the perspectives of inflammation, oxidative stress, metabolism and hormone secretion. We highlight the underlying molecular biological mechanisms linking COPD to cognitive dysfunction, with a focus on inflammation and oxidative stress. Finally, we discuss current therapeutic approaches for COPD and/or cognitive dysfunction associated with other neurological disorders, aiming to identify potential adjunctive treatments.

## 2. The association of COPD with brain disorders

Cognitive impairment is one of the common complications of COPD. There is growing evidence that impaired lung health, including COPD, may be associated with an increased risk of dementia or worsening cognitive abilities^39^, ^40^. A meta-analysis of 428030 individuals from the United Kingdom, the United States, and China found that people with COPD had a significantly higher risk of developing dementia or cognitive impairment^41^. Similarly, another meta-analysis involving 39392 COPD patients worldwide confirmed that COPD is associated with an elevated risk of dementia^42^.

Theoretically, COPD may increase dementia risk through multiple pathways, including oxidative stress, tissue hypoxemia, inactivity, and systemic inflammation^43^, ^44^. Among these, oxidative stress caused by systemic inflammation and hypoxia is considered a major contributor to dementia. Furthermore, hypoxemia in the context of obstructive sleep apnea has also been linked to an increased risk of dementia^45^. Taken together, chronic hypoxia, hypercapnia, and increased inflammatory cytokines in COPD patients can lead to brain damage, including neuronal dysfunction, structural changes and reduced white matter integrity, thereby exacerbating the development of degenerative brain disease.

A population-based study with over 25 years of follow-up found that middle-aged individuals with COPD or asthma had nearly twice the risk of developing dementia in later life^41^. These suggest that COPD may serve as an independent predictor of dementia. Broadly speaking, dementia encompasses a range of brain disorders, including Parkinson's disease (PD), Alzheimer's disease (AD), vascular dementia, and mixed dementia, with PD and AD being the most common types. On one hand, serum levels of Aβ40, Aβ42 and total Aβ, key players in the progression of AD, are significantly increased in COPD patients. On the other hand, frontal lobe defects and behavioral disorders occur more frequently in patients with both AD and COPD than in those with AD alone^46^. These indicate that cognitively normal patients with COPD may develop pathological changes associated with AD, and COPD may contribute to AD-type pathogenesis^47^, ^48^.

PD is characterized by the persistent loss of striatal dopaminergic neurons in the substantia nigra compacta, leading to striatal dopamine deficiency. The presence of activated microglia or elevated levels of inflammatory cytokines in the substantia nigra, striatum, and serum may play a role in PD development^49^-^52^. As previously discussed, COPD is an inflammatory disease and it may contribute to PD by inducing neuroinflammation and subsequent dopaminergic neuronal death^53^. Thus, inflammation in patients with COPD may be closely related to the development of PD.

Depression is another common comorbidity of COPD, with up to 55% of patients diagnosed with anxiety and/or depression^54^, ^55^. As the disease progresses, the mortality rate among COPD patients with depressive symptoms is significantly higher than among those without depression^56^. There is a bidirectional association between COPD and depression: COPD increases the risk of depression progression, while depression is associated with worse COPD outcomes^57^. Previous studies have suggested that many neurological issues in COPD patients, particularly mood disorders, may stem from the disease experience itself. For example, dyspnea (a core symptom of COPD) and the inability to perform daily activities may lead to depression and anxiety^54^.

However, recent studies have revealed that patients with depression exhibit significantly elevated levels of pro-inflammatory cytokines in their plasma and (or) serum. This has led to the "depression cytokine hypothesis", which posits that depressive symptoms may arise from direct inflammation-related pathways. Key cytokines implicated in depression include IL-1, IL-6, TNF-α and IFN-γ^58^, ^59^. Given that inflammation plays a well-established role in COPD pathogenesis, which is characterized by high levels of inflammatory mediators such as TNF-α, IFN-γ, IL-4, and IL-10^60^. Thus, it is plausible that serum biomarker levels may link COPD and depression. These cytokines could serve as risk factors for depressive mood in COPD patients, increasing the likelihood of depression^61^. Therefore, depression in COPD patients should not be viewed solely as a psychological distress but rather as a condition with potential inflammatory underpinnings.

In summary, COPD is closely associated with various brain disorders (Table 1), suggesting a potential lung-brain connection. COPD comorbidities should be anticipated as a common clinical challenge. Consequently, treatment strategies for COPD should evolve from focusing solely on pulmonary function recovery to addressing its comorbidities simul-taneously^62^.

## 3. The lung-brain axis: a link between COPD and neurological dysfunction and potential mechanism of COPD-NCDs

The mechanisms underlying COPD-NCDs are multifaceted. One potential pathway involves CS, which may directly cause neurocognitive disorders by damaging the vascular wall and altering endothelial function^63^. Additionally, respiratory insufficiency in COPD is strongly associated with hypoxemia and hypercapnia, both of which are linked to pulmonary airway inflammation. This inflammation can lead to insufficient oxygen levels in the blood (hypoxemia) and ultimately result in hypoxia^64^. Key drivers of this hypoxia include progressive airflow limitation and ventilation/perfusion mismatch caused by emphysema^44^. Furthermore, the prolonged experience of illness can lead to emotional disorders in patients, such as anxiety and depression.

**Figure 1:**
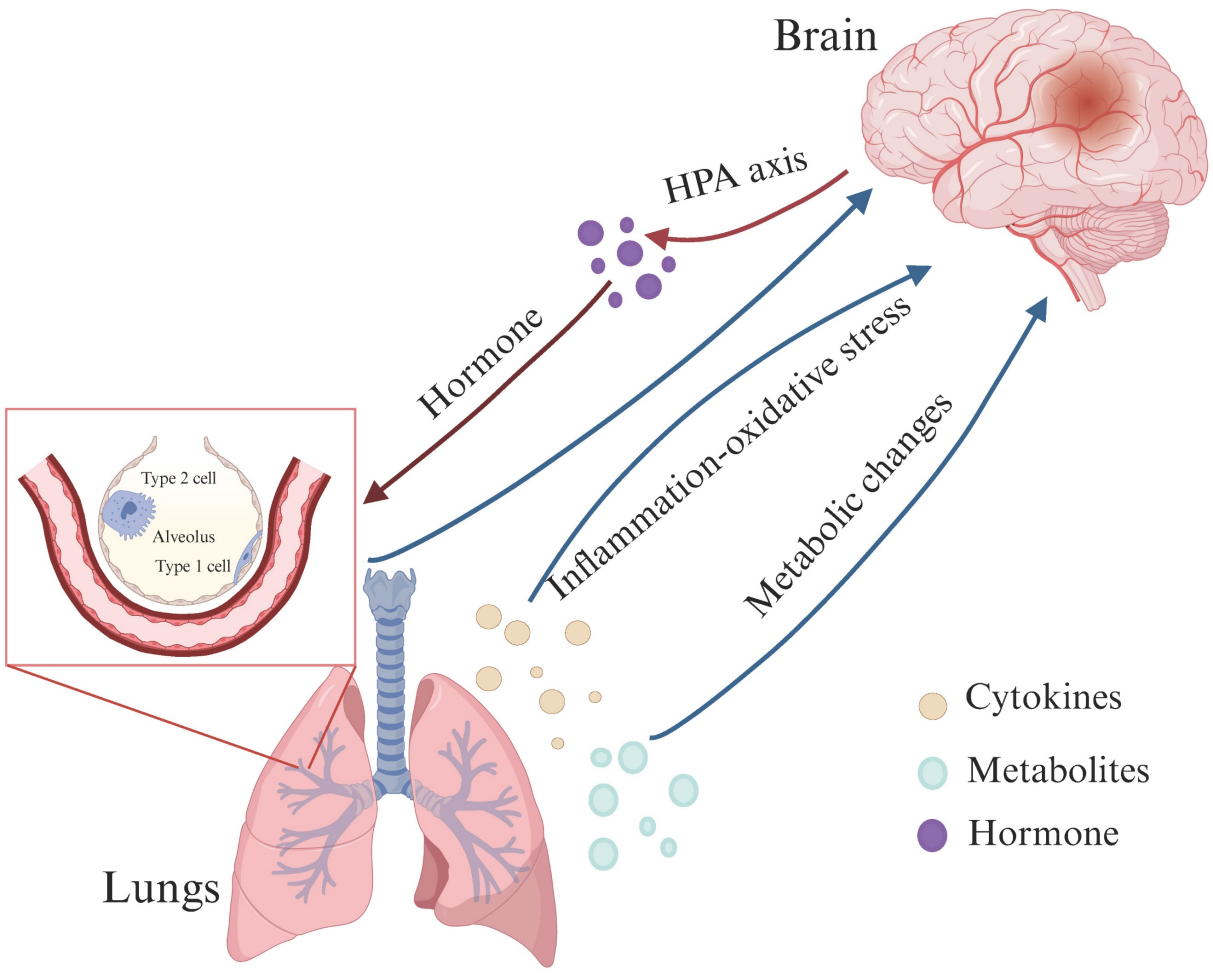
** The lung-brain axis: possible links between COPD and neurological dysfunction.** The lung-brain axis is believed to operate bidirectionally and this intricate interplay is facilitated by a complex network involving neuronal, inflammatory, immune, and neuroendocrine signaling pathways (HPA axis). (Created with Biorender.com).

Recent studies have also pointed out that a lung-brain axis may be established between lung and brain through blood circulation. Inflammatory factors, metabolites and hormones may affect brain structure and function through this lung-brain axis, thereby causing neurocognitive disorders^33^, ^65^. This axis is believed to operate bidirectionally, with neurological dysfunction and lung-related infections influencing each other through a complex network of neuronal, inflammatory, immune, and neuroendocrine signaling pathways (Figure 1)^66^.

For example, one study demonstrated that anesthesia/surgery increased the levels of Tau phosphorylated at threonine 217 in the blood by promoting its production and release. This phosphorylated Tau binds to B cells, which may then enter the brain and increase neuronal excitability, leading to postoperative delirium-like behaviors in aged mice^67^. In another study, Annexin A5 was shown to reduce infarct areas and improve general neurologic function following cerebral ischemia. Increased levels of Annexin A5, potentially derived from lung tissue, were found to permeate the BBB and provide neuroprotection^68^. These findings suggest that such biomarkers or factors could serve as mediators or therapeutic targets to improve lung and brain health in the future.

### 3.1 Lung-brain axis associated with Metabolic changes

It is well documented that bacteria in the gastrointestinal (GI) tract can activate neural pathways and CNS signaling systems, thereby influencing brain function and increasing the risk of diseases such as anxiety and depression. This interaction between microbial metabolites and brain is known as the gut-brain axis. For example, indole-3-propionic acid, a metabolite produced by gut bacteria, has been shown to promote sensory axon regeneration and functional recovery through an immune-mediated mechanism^69^. The respiratory tract is one of the primary entry points for microorganisms into the human body^70^, making it a key role in shaping the composition of the microbiota. Respiratory diseases can alter the local microbial composition of the lungs, and an imbalance in the lung microbiome is associated with inflammation, immune response, and clinical deterioration. In COPD, the diversity of the lung microbiome is influenced by factors such as treatment type, disease severity and inflammation. For example, *Actinomyces, Actinobacillus, Megasphaera, Selenomonas* and *Corynebacterium* are significantly more abundant in COPD patients compared to healthy individuals^71^. Another study found that *Pseudomonas* plays a role during COPD exacerbations, while *Streptococcus* and *Rothia* may help prevent exacerbations^72^.

An increasing amount of experimental and epidemiological evidence has emphasized the important communication between the microbiota of the lungs and the intestines, known as the "gut-lung axis"^73^. The gut microbiota can resist viral respiratory infections and act as key regulators of respiratory capacity, highlighting the bidirectional nature of this axis^74^. Disruption of this axis, such as through exposure of the lungs to zinc oxide nanoparticles (ZnONPs), can cause dysbiosis and subsequently lead to harmful effects on the nervous system^75^. Furthermore, recent studies have shown a tight link between the lung microbiome and the brain immune reactivity: local lung microbes continuously send signals to microglia, which in turn regulate immune responses. In a rat model, dysregulation of the lung microbiome significantly increases susceptibility to multiple sclerosis, an autoimmune disease of the CNS^65^. Additionally, microbiome assays in a lipopolysaccharide (LPS)-induced mouse model of experimental severe pneumonia have revealed similarities between the bacterial species in the brain and those in the lungs, suggesting that bacteria in the brain may originate from the lungs during pneumonia^76^. This raises the possibility that the link between lung microbiota and the immune response in the brain could be exploited to treat COPD-NCDs. For example, treatment could involve the topical application of probiotics or targeted antibiotics.

Increasing evidence suggests that such an axis may also exist between the lung and brain, influencing brain function through metabolic regulation^77^. For instance, peripheral delivery of mesenchymal stem cells can activate vagal sensory neurons innervating the lungs, induce the release of 5-hydroxytryptamine in the dorsal raphe nucleus, and further alleviate depression- and anxiety-like behaviors^78^. Studies using localized proton magnetic resonance spectroscopy have revealed significant alterations in brain metabolism in symptomatic COPD patients, including reduced choline levels, which are associated with memory dysfunction. These findings provide a neurochemical basis for brain dysfunction in COPD patients^79^. Clinical studies have also shown that a reduction in the diversity of bacterial microbial communities in the lungs leads to increased alveolar catecholamine concentrations^80^. While high levels of catecholamines can have neurotoxic effects, their metabolites circulate throughout the body and are linked to neurological diseases such as PD and depression. In addition, impaired lung function may be associated with decreased glucose metabolism in different brain regions. For example, COPD-induced lung dysfunction causes cerebral hypoperfusion, which further reduces brain glucose uptake. Decreased cerebral glucose metabolism is closely associated with cognitive impairment^81^. Therefore, further exploration of these mechanisms in animal models could provide new insights and therapeutic targets for COPD-NCDs.

Recent studies have proposed the "triple-hit" hypothesis, which suggests that lung injury triggers a cascade of events, including immune disorders, inflammatory responses, and microbiota changes. These events activate the "lung-gut axis", leading to the "triple-hit" that contributes to the development or worsening of cognitive deficits^82^. Gut microbiota therapy has shown promise in altering the inflammatory processes along the "gut-lung-brain axis", alleviating lung injury, and therapeutically regulating brain function and behavior. These findings offer new insights for treating cognitive deficits associated with lung injury caused by gut dysbiosis.

### 3.2 The axis associated with inflammation-oxidative stress between lung and brain

Peripheral inflammation, such as GI inflammation, rheumatoid arthritis, and COPD, can lead to neuroinflammation through various mechanisms. Among the brain regions most affected by peripheral inflammation are the hippocampus, cortex, amygdala and hypothalamus^83^. Potential mechanisms include destruction of the BBB, activation of glial cells associated with systemic immune activation, and effects on the autonomic nervous system through the organ-brain axis 84-89. Neuroinflammation can cause neuronal tissue damage and significantly impact neuropsychiatric symptoms, including spatial memory impairment, cognitive dysfunction, anxiety and depression.

COPD is characterized by chronic inflammation in the lungs, airways and bronchoalveolar lavage fluid. In the context of neuroinflammation, immune-related factors and BBB disruption are widely recognized as fundamental causes^84^, ^90^. When exposed to harmful stimuli such as CS, macrophages and neutrophils release inflammatory mediators including TNFα^91^, IL-1β^92^, MCP-1^93^, and CCL3^94^. These mediators activate nuclear factor-κB (NF-κB), prolonging the inflammatory response and promoting the release of protease (Table 2)^95^. Inflammatory factors can also disrupt tight junctions (TJs) in brain endothelial cells, increasing BBB permeability^96^.

Additionally, these inflammatory cells are a significant source of ROS, contributing to oxidative stress in the lungs. When exposed to CS, immune cells are activated to produce ROS and RNS through mitochondrial dysfunction^97^. Levels of ROS and RNS are elevated in COPD patients compared to healthy individuals^6^, ^98^, ^99^, while antioxidants such as glutathione peroxidase, catalase, and superoxide dismutase are reduced^100^. Oxidative stress occurs when the production of reactive substances and free radicals overwhelms the body's antioxidant defenses. This stress promotes apoptosis in the lung tissue, alveolar epithelial damage, mucus hypersecretion, oxidative inactivation of surfactants and antiproteases, further exacerbating COPD symptoms. Neurons, which rely heavily on mitochondrial respiration for energy, are particularly vulnerable to oxidative stress. High levels of ROS in neurons lead to the production of toxic peroxidized lipids, perpetuating oxidative damage and contributing to neurocognitive disorders in COPD^36^, ^101^. During COPD exacerbations, almost all markers of oxidative stress, including malondialdehyde, superoxide (O^2-^) and hydrogen peroxide (H_2_O_2_), are further elevated^102^. Moreover, oxidative stress can activate resident lung cells, such as alveolar macrophages and epithelial cells, resulting in the production of chemotactic molecules. These molecules recruit additional inflammatory cells, including lymphocytes, monocytes, and neutrophils, into the lungs. Thus, oxidative stress and inflammatory responses mutually reinforce each other, creating a vicious cycle that perpetuates lung and brain damage.

**Figure 2:**
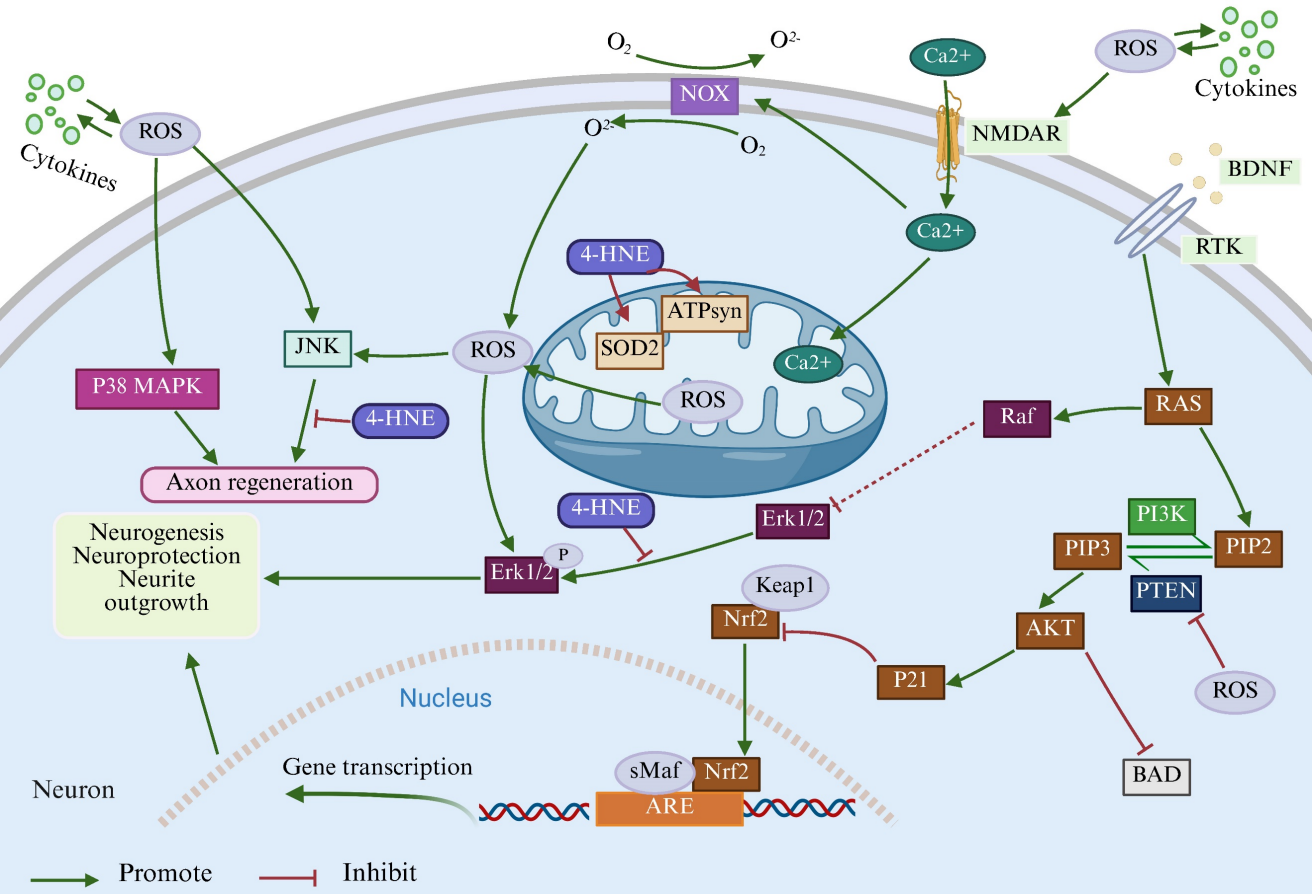
** Possible signaling pathways in inflammatory-oxidative stress mediated neural regeneration.** CNS injury induces cytokines to interact with ROS and activation of NMDAR leads to calcium efflux, thereby further contributing to intracellular ROS accumulation through the action of NOX. Intracellular ROS inactivates PTEN and leads to the accumulation of PIP3, which directs Akt to the plasma membrane and promotes Akt activation and neurogenesis. In addition, Akt activates p21, thereby disrupting the interaction between Nrf2 and its inhibitor Keap1 and promoting Nrf2 stabilization for detoxification and neuroprotection against excessive ROS action. RTK are up-regulated in response to CNS injury and activate ERK1/2 signaling pathway upon binding to BDNF. All of the above events promote neurogenesis, neuroprotection and synaptic maturation. Finally, ROS induces JNK p38 MAPK, whose synergistic action is essential for debris removal and axonal regeneration. However, high levels of oxidative stress can trigger peroxidation and the formation of 4-HNE lipids, which may adversely affect nerve regeneration. (BDNF - brain-derived neurotrophic factor; ERK1/2 - extracellular signal-regulated protein kinase; 4-HNE - 4-hydroxy-2-nonenal; JNK - c-jun N-terminal kinase; MAPK - mitogen-activated protein kinases; NMDAR - N-methyl-d-aspartate receptor; NOX - NADPH oxidase; Nrf2 - nuclear factor erythroid 2 like 2; PI3K - phosphatidylinositol 3-kinase; PIP2 - phosphatidylinositol (4,5)-bisphosphate; PIP3 - phosphatidylinositol 3,4,5-trisphosphate; PTEN - phosphatase and tensin homolog; RTK - receptor tyrosine kinase) (Created with Biorender.com).

From a deeper perspective, inflammation-oxidative stress is involved in neuronal development, functional polarization, connectivity and plasticity by regulating multiple signaling pathways, including Nrf2, PI3K/AKT, JNK, Hedgehog, etc^103^ (Figure 2). For example, nicotinamide adenine dinucleotide phosphate (NADPH) oxidases (NOX)-produced ROS can regulate not only the stemness of neural progenitor cells through PI3K/AKT signaling pathway but also axon path finding via the Hedgehog pathway^104^. By promoting oxidative stress, NOX4 induces the production of the lipid peroxidation end-products, such as 4-hydroxynonenal (4-HNE) and malondialdehyde, and ultimately mediating ferroptosis-dependent cytotoxicity of astrocytes in AD^105^. Furthermore, 4-HNE has been shown to play a role in synaptogenesis during CNS development and neuronal function in the mature brain^106^. 4-HNE can bind to macromolecules altering their structure and function, including modification of ATP synthase or SOD2, which triggers excessive ROS generation and mitochondrial dysfunction, thereby promoting neurodegeneration^107^. The production of 4-HNE after CNS trauma may promote ROS generation, leading to sustained JNK activation^108^. Thus, increased inflammation-oxidative stress and down-regulated antioxidants may be key drivers of neurocognitive deficits, including those associated with COPD.

### 3.3 The hypothalamic-pituitary-adrenal (HPA) axis activation

The HPA axis is a neuro-endocrine "hub" that coordinates physiological responses to external stimuli. HPA axis dysfunction is associated with a number of mental health conditions, particularly anxiety and depressive disorders^109^, ^110^. Neurons in the CNS are capable of releasing neuroendocrine factors such as corticotropin-releasing hormone (CRH) and glucocorticoid (GC) in response to stress or circadian rhythm stimuli^111^. GC, a key component of the HPA axis, not only regulates blood glucose but also exerts immunosuppressive effects. These effects are mainly mediated through the tethering of the GC receptor to transcription factors (e.g., NF-κB and AP-1), which reduces the expression of pro-inflammatory genes^112^. Interestingly, GC exhibit a dual role in immune regulation: they have 'permissive' (that is, immunostimulatory) effects at low concentrations and suppressive effects at high concentrations^113^, ^114^.

We discussed earlier that inflammation in the lungs can trigger a systemic inflammatory response in the brain, leading to brain damage. In turn, immunosuppression resulting from CNS injury increases the risk of systemic infections, such as pneumonia and urinary tract infections^115^. Interestingly, children with asthma often develop adrenal insufficiency^116^, suggesting a potential link between lung inflammation and HPA axis dysfunction.

There is increasing evidence that the CNS and lungs interact bidirectionally through the HPA axis, which regulates the neuroinflammatory state in a dynamic manner^117^, ^118^. Multiple hormones within this axis, including CRH, adrenocorticotropic hormone (ACTH), GC, and mineral corticoid, play important roles in the central inflammatory response by binding to their corresponding receptors.

First, brain injury may activate the HPA axis and induce GC secretion^119^. GC is essential for normal lung development, regulating key events such as morphological changes, lung maturation, and surfactant synthesis in type II epithelial cells^120^. Second, CRH can increase bacterial adhesion and expression of inflammation regulatory genes in *streptococcus pneumoniae*, highlighting its role in bacteria-related inflammatory responses^121^. Additionally, studies on CRH-Angiotensin-converting enzyme 2 (ACE2) KI (ACE2 KI mice are genetically engineered to overexpress ACE2 under the ROSA26 promoter, with Cre-LoxP-mediated restriction to CRH-synthesizing cells) and wild type (WT) mice have shown that overexpression of ACE2 in CRH cells (derived from CRH-ACE2KI mice) inhibits HPA axis activity by decreasing CRH synthesis, resulting in reduced anxiety-like behavior^122^. CRH receptors (CRH-R1 and CRH-R2) are also present in the lungs, and CRH-R antagonists show promise as interventions for eczema, asthma, and urticaria.

Due to their anti-inflammatory and immunosuppressive properties, corticosteroids are a clinical option for COPD patients. However, controlled dosing is essential to minimize the risk of HPA axis suppression^123^. Studies have shown that basal serum corticosterone levels are significantly reduced in COPD^124^, possibly due to stress-induced dysregulation of the HPA axis or excessive negative feedback of the HPA axis. Robitussin co-administration counteracts the stimulatory effect of COPD on basal circulating corticosterone levels, suggesting that suppression of inflammation and oxidative stress may help prevent excessive activation of the HPA axis. Increased lung inflammation and pulmonary dysfunction in COPD are strongly associated with social-cognitive memory deficits and neophobia in new environments^125^. Therefore, further studies on the HPA axis are needed to elucidate the mechanisms underlying COPD-NCDs.

## 4. Brain damage caused by COPD due to the presence of the lung-brain axis

**Figure 3:**
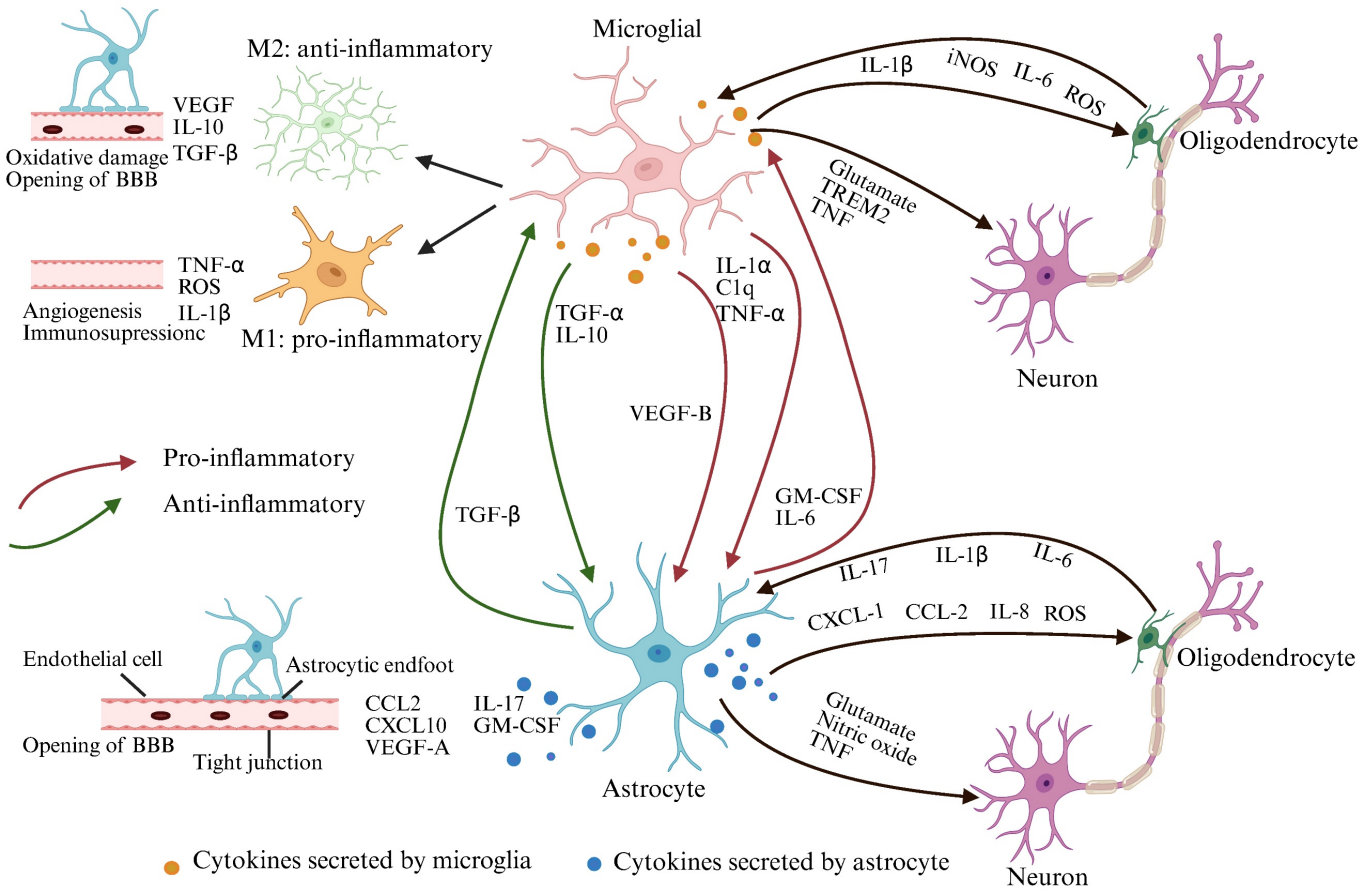
** COPD-NCDs may cause altered glial cell function, BBB permeability, and neuronal dysfunction.** Bidirectional communication between astrocytes and microglia regulates their responses during CNS inflammation. Upon activation, microglia and astrocytes release neurotoxic NO, glutamate, or downregulate extracellular neurotransmitter uptake, respectively, ultimately leading to neuronal and oligodendrocyte death. Microglia and astrocytes also control oligodendrocyte recruitment by secreting a variety of cytokines. Activated microglia can be polarized into M1/M2 phenotypes under different conditions. Protrusions of M1-type microglia become larger in diameter and present an amoebic state, which have neuroinflammatory effects, while M2-type microglia have significantly more branching and longitudinally extended protrusions, executing an anti-inflammatory effect and having a neuroprotective function. Interactions of activated microglia with astrocytes and endothelial cells increase BBB permeability, whereas bidirectional communication between astrocytes and peripheral immune cells enhances CNS inflammation and leads to disease progression (Created with Biorender.com).

The connection between smoking and neurological complications has been demonstrated in diseases such as stroke, AD, and multiple sclerosis^126^-^128^. However, the precise mechanisms underpinning COPD-related cognitive impairment remain largely undefined. Studies have shown that elevated markers of lung inflammation and oxidative stress in COPD may extend beyond the lungs, triggering inflammation and causing damage to the brain. This process is reflected in altered BBB permeability, brain atrophy, activation of brain immune cells, and neuronal damage or loss, ultimately leading to cognitive deficits (Figure 3). Therefore, our focus is on exploring the potential mechanisms by which inflammation and oxidative stress contribute to COPD-NCDs.

### 4.1 Altered glial cell function

Astrocytes and other glial cells such as microglia, create and maintain a highly controlled microenvironment essential for efficient neuronal function within the CNS^129^. Glial cells were initially thought to merely provide nutritional support to neurons. However, it is now clear that tightly intertwined neuronal-glial networks are crucial for optimal CNS function^130^-^132^. Emerging evidence from preclinical and clinical studies highlights the role of astrocytes and microglia in neurocognitive functions. For example, Zhou and colleagues proposed that astrocytes play a key role in maintaining the excitation-inhibition balance and the neurotrophic status of local networks responsible for anxiety-like behavior. This may modulate synaptic activity, thereby influencing cognition^133^.

However, CS exposure can inhibit hippocampal astrocyte density, reduce the expression of synaptosomes and shorten dendritic spines^134^. Nicotine, a component of CS, can disrupt astrocyte-neuron interactions through direct or indirect mechanisms. Directly, nicotine binds to nicotinic acetylcholine receptors on astrocytes^135^. Indirectly, nicotine may alter astrocyte function by increasing local dopamine concentrations. For instance, dopamine can trigger CCCTC-binding factor-dependent morphological and genomic remodeling of astrocytes^136^.

In the brain, microglia are the primary immune cells, accounting for about 75%-80% of all brain immune cells^137^. They play a dual role in neurodegenerative diseases. On the one hand, activated microglia help clear neuronal debris through phagocytosis, on the other hand, they contribute to disease progression by releasing molecules that induce a neuroinflammatory state^138^-^140^. M1-activated microglia produce pro-inflammatory mediators^141^, increase indoleamine 2, 3-dioxygenase (IDO) activity, and shift tryptophan metabolism toward the kynurenine pathway, leading to neuronal death^142^. In addition, microglia secrete IL-1α, TNF-α and complement 1q (C1q), which can induce astrocytes to produce neurotoxic factors, reduce phagocytic activity and decrease the expression of neurotrophic factors^143^.

More importantly, CS exposure significantly affects hippocampal microglia volume, impairing working memory maintenance. These microglia exhibit a more activated morphology^134^. Chronic activation of microglia leads to loss of neurons and axons, resulting in neurocognitive deficits. It has been demonstrated that CS exposure increases the density of astrocytes and microglia in the suprachiasmatic nucleus (SCN) region of the hypothalamus, potentially impairing social recognition memory and increasing fear of new environments. Treatment with the antioxidant apocynin restores morphology of microglia but does not affect astrocyte levels^125^.

### 4.2 BBB permeability changes

The BBB is composed of capillary wall endothelial cells, pericytes, and astrocytes^144^. It plays a crucial role in regulating the transport of molecules into and out of the brain, protecting it from harmful substances and pathogens under healthy conditions^145^. Microglia are strongly associated with BBB permeability and play a dual role in maintaining BBB integrity during inflammation. Initially, microglia protect BBB integrity by interacting with cerebral blood vessels. However, further prolonged inflammation shifts microglia to a more active phenotype, which can phagocytize astrocyte end-feet and impair BBB permeability^146^.

During neuroinflammation, activated microglia produce IL-1β, which induces astrocytes to release vascular endothelial growth factor-A (VEGF-A). VEGF-A, in turn, downregulates TJ proteins such as claudin-5 (Cldn5) and occluding (Ocln) in endothelial cells via endothelial nitric oxide synthase (eNOS)-dependent mechanisms. This disrupts the TJs and increases BBB permeability^143^. These findings were further supported by a CS and LPS-induced murine model of COPD, which showed reduced expression of Cldn5 and Ocln in the cerebral microvasculature. Additionally, toxic components of CS can enter the bloodstream and compromise BBB integrity^147^. Both CS and e-cigarettes have been shown to reduce key TJ proteins including Ocln^148^ and ZO-1^149^, strongly suggesting that smoking is associated with loss of BBB integrity.

In the course of COPD disease, pro-inflammatory cytokines, ROS, CRP may disrupt the regulation of TJs in brain endothelial cells, increasing BBB permeability, damaging brain cells, and promoting atherosclerosis in both the anterior and internal cerebral arteries^96^, ^150^, ^151^. These findings suggest that neuroinflammation in brain regions associated with cognitive function can activate microglia, injure astrocytes, and affect the expression of key TJ proteins, ultimately increasing BBB permeability.

The BBB has mechanisms to prevent leakage when exposed to inflammatory stimuli^152^. However, increased permeability allows toxic circulating molecules, such as inflammatory cytokines, ions, and immune cells, to enter the brain microenvironment, further compromising BBB integrity^84^. Collectively, these studies emphasize that COPD causes pulmonary/peripheral inflammation to “spill-over” into the CNS, increasing BBB permeability. However, the underlying mechanisms of COPD-NCDs need to be explored in greater depth.

### 4.3 Neuronal dysfunction

Due to the existence of the forementioned lung-brain axis, COPD patients experience brain damage caused by inflammation and oxidative stress. On the one hand, neurons, as post-mitotic cells, are exposed to ROS for longer periods than other dividing cells in the brain. This makes adult neurogenesis highly dependent on tightly regulated redox signaling pathways. CS, the most important predisposing factor of COPD, can disrupt the oxidative balance, altering oxidative homeostasis in the brain and periphery, and significantly reduce the number of DCX-positive immature neurons^153^. Neuronal cells contain the antioxidant glutathione (GSH), which regulates excessive ROS^154^, ^155^. The loss of GSH can cause oxidative damage, leading to dendritic damage, neuronal death and cognitive impairments^156^. Further studies have confirmed that neuronal GSH depletion is linked to the downregulation of excitatory amino acid transporter 3 (EAAT3), resulting from lipid peroxidation-induced neuronal membrane dysfunction^157^. In BALB/c mice, CS exposure generates ROS-induced oxidative stress and significantly reduces antioxidant defense in the brain by depleting GSH, GPX and superoxide dismutase. On the other hand, CS-induced neuroinflammation is markedly increased, affecting the expression of synaptophysin, reducing mature dendritic spines, and compromising synaptic integrity^134^, ^158^. The formation of functional synapses between neurons is essential for establishing neural circuits that support learning and memory^159^. There is no doubt that synaptic and dendritic spine damage caused by COPD neuroinflammation significantly impairs neurocognitive function and memory.

## 5. Potential treatments for COPD-NCDs

At present, there is a lack of effective comprehensive treatments for cognitive impairment in COPD individuals. Identifying medications that can not only alleviate COPD symptoms but also mitigate and reverse cognitive dysfunction in COPD patients is crucial for disease management. As previously discussed, inflammation and oxidative stress are likely key contributors to cognitive dysfunction in COPD patients^160^-^162^. Therefore, anti-inflammatory and anti-oxidative stress therapies targeting COPD and/or cognitive dysfunction associated with other neurological conditions may serve as complementary treatments (Table 3)^163^.

### 5.1 Anti-inflammatory treatment of COPD-NCDs

The increased prevalence of cognitive impairment in COPD is associated with the stability of COPD symptoms, the duration of pathology, and the severity of respiratory symptoms^163^. In general, persistent systemic inflammation in the lungs and localized neuroinflammation in the brain, are significant triggers of depression and cognitive deficits^164^. Elevated inflammatory markers in the lungs of patients with COPD can lead to inflammation and damage in the brain, resulting in cognitive deficits^64^. Consequently, anti-inflammatory treatments for COPD patients may not only alleviate the respiratory symptoms but also have therapeutic effects on cognitive dysfunction. However, anti-inflammatory drugs may alter the natural course of COPD progression and potentially negatively impact the nervous system.

For instance, corticosteroids are commonly used to reduce lung inflammation and improve airway obstruction in COPD^165^. However, airway inflammation in COPD is relatively resistant to corticosteroid therapy and overcoming corticosteroid resistance remains a challenge. Importantly, GCs (a class of corticosteroids) have been shown to negatively affect cognition, with subjects experiencing impaired memory after taking GCs. Resistance to the anti-inflammatory effects of steroids is a major barrier to effective COPD treatment. Therefore, developing broad-spectrum anti-inflammatory therapies as alternatives to corticosteroids is a promising strategy. New anti-inflammatory agents are needed to address cognitive impairment in COPD^166^, ^167^.

Beyond corticosteroids, other anti-inflammatory agents have been studied in human and animal models. Roflumilast, for instance, not only reduces airway inflammation and bronchoconstriction in patients with COPD^168^, but also shows potential as a memory enhancer at low doses^169^. It may exert neuroprotective effects in early brain injury after subarachnoid hemorrhage through anti-inflammatory mechanisms^170^. It can also reduce neuroin-flammation and cognitive deficits in sporadic AD^171^. However, roflumilast has a narrow therapeutic window and can cause GI and CNS side effects, limiting its widespread use. Roflumilast is the first anti-inflammatory drug approved for COPD treatment^172^. Despite its side effects, such as nausea and vomiting in 5% of patients at the approved dose of 500 μg, low doses (100-250 μg) have shown cognitive-enhancing effects in healthy adults, older adults, individuals with mild cognitive impairment, and patients with schizophrenia^173^. Clinical trials are currently investigating its potential benefits for early AD, post-stroke cognitive impairment and fragile X syndrome. Overall, roflumilast demonstrates promising effects on cognitive performance.

Another potential treatment is formoterol, a long-acting β2 adrenergic receptor agonist used to relieve respiratory symptoms in asthma and COPD. Formoterol has been shown to improve cognitive function by enhancing synaptic density in the hippocampus and increasing the neuronal complexity of newly formed dentate granule neurons in mice^174^. Therefore, both roflumilast and formoterol may be promising candidates for treating COPD-NCDs. Additionally, other anti-inflammatory agents, such as sodium-glucose cotransporter 2 inhibitors (SGLT2i)^175^, celecoxib^176^, aspirin^177^, and N-3 polyunsaturated fatty acids^178^, may also play prominent roles. For instance, SGLT2i has demonstrated pleiotropic benefits for cognitive disorders, epilepsy, movement disorders, and stroke^175^. Their anti-inflammatory and anti-apoptotic effects can reduce BBB leakage, attenuate microglia activation, and promote myelin remodeling, thereby combating neuroinflammation in various diseases^179^. Therefore, anti-inflammatory drugs with neuroprotective properties may have profound effects on COPD-NCDs.

### 5.2 Antioxidants for COPD-NCDs

Oxidative stress, resulting from an imbalance between oxidants and antioxidants, plays a crucial role in the pathogenesis of COPD^180^. Studies have shown that mediators of oxidative stress may lead to a sustained systemic inflammatory response, resulting in brain damage and eventually contributing to COPD-NCDs^181^, ^182^. Consequently, antioxidant therapy not only attenuates oxidative stress in the lungs but also ameliorates COPD-NCDs.

Ebselen, for instance, has demonstrated protective effects in conditions characterized by oxidative stress, such as diabetes-related atherosclerosis, ischemia/reperfusion injury, and pulmonary inflammation^183^-^186^. It significantly reduces CS-induced lung inflammation and vascular oxidative stress, while restoring vascular endothelial function^187^. In addition, Ebselen has been used in acute ischemic stroke^188^, ^189^ and has been shown to completely block CS-induced impairment of working memory and spatial memory by restoring synaptophysin expression^36^.

Another antioxidant, N-acetyl-L-cysteine (NAC), has been shown to reduce oxidative load in the airways of patients with stable COPD^190^. NAC also reverses depression, anhedonia, and anxiety-like behaviors in a neonatal clomipramine model of depression^190^ by restoring AChE activity and rescuing the loss of synaptic plasticity. Furthermore, NAC prevents STZ-induced cognitive dysfunction^191^, ^192^. Other antioxidants, such as Apocynin^193^, the BET/BRD inhibitor JQ1^194^, ^195^, and Vitamin C^196^-^199^, have also been reported to exert antioxidant effects through different mechanisms, and require further investigation.

To date, most treatments for COPD are in preclinical or early clinical development, while studies for COPD-NCDs have been limited to animal models. Therefore, the outcomes of future clinical trials targeting COPD-NCDs are highly anticipated. Beyond pharmacological approaches, stem cell-based regenerative therapies may indirectly modulate inflammation in COPD and COPD-NCDs without requiring precise endotype precision. Moving forward, clinical studies are needed to improve the treatment of this challenging condition.

## 6. Conclusions

COPD-NCDs represent an emerging area of research. Despite their high prevalence, neurocognitive comorbidities are often under-diagnosed and inadequately treated, leading to poor patient outcomes. This is primarily due to the lack of a systematic understanding of the pathogenic mechanisms, disease progression, and effective treatments for COPD-NCDs. From the perspective of the lung-brain axis, we have summarized the factors and mechanisms underlying the interaction between the lungs and brain in the pathogenesis of COPD-NCDs. A comprehensive understanding of neurological dysfunction caused by COPD through the lung-brain axis may provide powerful clues or evidence for the precise diagnosis, personalized treatment, and prognosis of COPD-NCDs. Ultimately, aiding in the clinical implementation of tailored treatment plans. Improving inflammation, oxidative stress, and metabolic dysregulation will be essential for treating COPD-NCDs, while neuroprotective therapies may serve as effective adjuncts.

In conclusion, COPD can induce BBB permeability changes, neuronal dysfunction, neuroglial alterations, and subsequent neurocognitive impairment through mechanisms involving oxidative stress, inflammatory factor spill-over, metabolic shifts, and pathways within the lung-brain axis, such as the HPA axis. Neuroprotective anti-inflammatory drugs and antioxidants have shown promise as therapeutic targets for alleviating neurocognitive disorders associated with COPD.

## Figures and Tables

**Table 1:** The association of COPD with brain disorders.

Risk factors	Mental and neurological complication	Pathological Features	References
Smoking Hypertension Hypoxaemia	Dementia	A reduction in volume and density of frontal grey matter	17 42 43
Smoking Age	Dementia AD	Abnormal static and dynamic local neural activity in the parahippocampal/hippocampal cortex	41
Smoking	AD Depression	Frontal deficits	45 46
Smoking Hypoxia	AD Dementia	Serum Amyloid-Beta Levels are Increased	47 48
Smoking Inflammation Age Hypoxia	PD	Microglia activation Elevated levels of inflammatory cytokines	44 49 50 51 52
Smoking Age	Depression Anxiety disorder	Activation of NLRP3 inflammasome and NF-κB pathways in the hippocampus	54 55
Smoking Hypogonadism Age	Depression	Low testosterone levels	55 56
Smoking	Depression	Elevated levels of inflammatory cytokines	57 58 59

**Table 2:** Neuropathological changes caused by the lung-brain axis associated with inflammatory-oxidative stress.

Animal Model	Inflammatory Factors in the Lungs	Inflammatory Factors in the Brain	Neuropathological changes	Pathways	References
Male BALB/c mice smoked for 24 weeks	TNF-α, IL-6, IL-1β, Cybb, NOX2, Mmp12	/	Increased astrocyte density	/	125
Male BALB/c mice smoked for 24 weeks	TNF, Ccl2, Cxcl1, Nox1/2	Itgam	Decreased number of microglia, shortened protrusion length, and altered astrocyte density	ZO-1 occludin, albumin, IgG	134
Male C57BL/6J mice smoked for 16 weeks	MCP-1, IL-1β, IL-6, TNF-α, IL-18	IL-1β, IL-6, TNF-α, IL-18	/	NLRP3, NF-κb, GR, Capase-1	55
Male C57BL/6J mice O_3_ for 13 weeks	/	Nlrp3, IL-1β	Microglia decreased	ChAT, Slc18a3, AChE	48
Male BALB/c mice smoked for 8 weeks	TNF-α Cxcl1/2, Ccl2 Mmp12, Mmp9 Gpx1, Nox1/2/4	TNF-α, Cxcl1, IL1β, Csf1, Mmp9, NOX1, GPx1, iNOS	The number of microglia in CA1 region did not change significantly, and the area of microglia increased	/	36
C20BL mouse anesthesia model	IL-1β, IL-6, TNF-α	IL-1β, IL-6, TNF-α	Increased numbers of microglia and astrocytes	TLR4	90

**Table 3:** Anti-inflammatory and antioxidant stress therapy for COPD-NCDs.

Treatment	Function	Mechanisms	Merit and Demerit	References
GCs (a class of corticosteroids)	Reduce lung inflammation; Improve airway obstruction	Mediating side effects; Mediating anti-inflammatory effects	Impaired memory capacity	165
Roflumilast (PDE inhibitors)	Reduce airway inflammation and bronchoconstriction; Reduce neuroinflammation and cognitive impairment	Decreased BBB permeability, levels of pro-inflammatory cytokines IL-1β, IL-6 and TNF-α, and neuronal apoptosis	GI adverse effects (nausea, vomiting, and GI reactions) and weight loss are common	168 169 170
Formoterol	Increase dendritic complexity and synaptic activity; Enhance cognitive function; Anti-inflammatory Dilatation of bronchial tubes	Regulation of Fgf2 gene expression	The ability to cross the BBB and improve cognitive function; Bronchodilator effect is dose-dependent	174
Sodium-glucose cotransporter protein 2 inhibitor (SGLT2i)	Reduce ROS; Reduce BBB leakage; Reduce microglia burden and acetylcholinesterase levels; Reduce carbon dioxide load	Acting on SGLT2 receptor	Reduction in cardiovascular risk factors; Augmentation of ketogenesis; Symptoms accompanying osmotic diuresis, pancreatitis and temporary hypoglycemic episodes	175
Celecoxib (COX-2 inhibitors)	Anti-inflammatory; Prevention of behavioral dysfunction	Inhibiting the expression of iNOS and COX-2 by inactivated of NF-κB	Prevention of early sAβ-induced neurotoxicity	176
Aspirin	Delay cognitive decline	It affects the inflammatory response by up-regulating PPARγ and down-regulating cyclooxygenase-1 and cyclooxygenase-2	Reduce hospitalization mortalityand shorten hospitalization time; Reduce the dependence on invasive mechanical ventilation	177
N-3 polyunsaturated fatty acids (N-3 PUFAs)	Anti-inflammatory	Binding to membrane phospholipids of inflammation-associated cells; Reduce neuroinflammation; Down-regulating PPAR and NFkB signaling pathway	/	178
Ebselen	Antioxidant stress; Block CS-induced impairment of working and spatial memory	Regulating synaptophysin expression without altering microglia activation	Attenuation of pulmonary inflammation; Decreasing in neutrophilic infiltration; Maintenance of synaptophysin density	187 188 189 35
N-acetyl-L-cysteine (NAC)	Antioxidant Stress; Alleviate depression	Blocking increased AChE activity and decreasing pTrkB and MnSOD levels	Improvement of cognitive function	190
Apocynin	Attenuate the deterioration of vascular endothelial function; Reduce vascular oxidative stress and injury; Reduce airway fibrosis and inflammation	Inhibition of NF-κB signaling pathway	/	193
BET/BRD inhibitors	Anti-inflammatory; Antioxidant Stress	Decreasing the expression of pro-inflammatory regulators Il-1b, Il-6, Tnfa, Ccl2, Nos2 and Ptgs2; Decreasing tau phosphorylation at the Ser396 site Increasing expression of Nrf2	Reducing cognitive dysfunction	194 195
Vitamin C	Antioxidant Stress	Activation of the Nrf2-ARE pathway to increase SOD-1 and GSTO1/2 expression	/	196 197 198 199
